# Psychoactive Substance Use and Problematic Internet Use as Predictors of Bullying and Cyberbullying Victimization

**DOI:** 10.1007/s11469-017-9809-0

**Published:** 2017-09-19

**Authors:** Ágnes Zsila, Gábor Orosz, Orsolya Király, Róbert Urbán, Adrienn Ujhelyi, Éva Jármi, Mark D. Griffiths, Zsuzsanna Elekes, Zsolt Demetrovics

**Affiliations:** 1 Doctoral School of Psychology, ELTE Eötvös Loránd University, Budapest, Hungary; 2 Institute of Psychology, ELTE Eötvös Loránd University, Budapest, Hungary; 3 Institute of Cognitive Neuroscience and Psychology, Hungarian Research Centre for Natural Sciences, Budapest, Hungary; 4 Psychology Department, Nottingham Trent University, Nottingham, UK; 5 International Gaming Research Unit, Psychology Department, Nottingham Trent University, 50 Shakespeare Street, Nottingham, NG1 4FQ UK; 6 Institute of Sociology and Social Policy, Corvinus University of Budapest, Budapest, Hungary

**Keywords:** Bullying, Cyberbullying, Victimization, Substance use, Problematic Internet use, Addiction

## Abstract

Research exploring the relationship between addictions and experiences of bullying suggests that problem behaviors may generally be associated with an increased risk of victimization. The aim of the present study was to examine the role of psychoactive substance use, excessive Internet use, and social support in both traditional offline bullying and online “cyberbullying” victimization in a nationally representative sample of adolescents (*N* = 6237; 51% male; *M*_age_ = 16.62 years, SD = 0.95). Results demonstrated that traditional bullying victimization was associated with cyberbullying victimization. Furthermore, psychoactive substance use and problematic Internet use predicted both traditional bullying and cyberbullying victimization. Finally, perceived social support was found to be an important protective factor against both traditional and cyberbullying victimization. However, psychoactive substance use and problematic Internet use accounted for only a small proportion of variance in victimization.

Bullying is defined as a repeated, aggressive act that is characterized by power imbalance, intentionality, and negative psychological consequences for both the perpetrator and the victim (Olweus 1999). Over the past few decades, the presence of modern technological devices (e.g., smartphones) in contemporary Internet-saturated lives has opened up a varied set of new experiences in addition to possible threats. For instance, aggressive behaviors have permeated into the domain of online communication and hence extended the scope of traditional bullying experiences. Cyberbullying has been defined as a repeated, hostile behavior intended to inflict harm via electronic devices (Patchin and Hinduja 2006; Tokunaga 2010) and has become a major concern in relation to youth well-being in developed countries throughout the world (Bauman and Bellmore 2015).

Due to the inconsistency in the definition, conceptualization, and assessment of traditional bullying and cyberbullying, relatively wide differences can be found in prevalence rates for both traditional bullying victimization (from 2.2 to 56.2% for traditional bullying and from 9 to 97.9% for cyberbullying) (Modecki et al. 2014; Perren et al. 2010; Pornari and Wood 2010; Slonje and Smith 2008). However, a recent meta-analysis by Modecki et al. (2014) indicated that the means of adolescent prevalence rates across 80 studies were 36% for traditional bullying victimization and 15% for cyberbullying victimization.

While the majority of studies did not find gender differences either in traditional bullying or cyberbullying victimization (e.g., Li 2007; Patchin and Hinduja 2006; Ybarra et al. 2007), a number of studies have demonstrated that girls are more likely than boys to be harassed online (e.g., DeHue et al. 2008; Kowalski and Limber 2007), whereas boys were at a greater risk than girls for being bullied at school (e.g., Boulton and Underwood 1992; Wang et al. 2009). However, relatively little attention has been paid to the role of school type in bullying experiences. One study reported that cyberbullying experiences were more prevalent in public schools than in private schools (Topçu et al. 2008).

Olweus (2013) indicated that 90% of cyberbullying victims were also involved in schoolyard bullying. However, research on the key features of traditional bullying and cyberbullying victimization suggests that the two phenomena have both convergent and divergent predictors (Casas et al. 2013). For instance, perceived school climate and the quality of peer interactions appear to predict victimization in both types of bullying (Casas et al. 2013), whereas risky Internet use shows a stronger association with cyberbullying than with traditional bullying (Casas et al. 2013; Erdur-Baker 2010).

Regarding Internet-related activities, prior studies have shown that the increased use of social networking sites is associated with cyberbullying victimization, particularly among girls (Mesch 2009; Sampasa-Kanyinga and Hamilton 2015). Furthermore, a recent study by Chang et al. (2015) identified online gaming as a risk factor for cyberbullying victimization. This study highlighted the importance of explorative research on specific Internet activities and platforms that may possibly be associated with an elevated risk of being bullied online. Maladaptive behaviors such as substance use (i.e., smoking cigarettes, drinking alcohol, and taking illicit drugs) (Litwiller and Brausch 2013; Luk et al. 2010) and school problems (e.g., detentions and suspensions) were associated with both types of bullying (Ybarra et al. 2007). Consequently, it has been suggested that antisocial problematic behaviors may generally increase the risk of victimization (Gámez-Guadix et al. 2013; Jessor 1991). In turn, social support has been identified as an important protective factor. Parental mediation has been associated with a decreased risk of cyber-victimization, particularly among boys (Mesch 2009; Wade and Beran 2011). Similarly, youth that have positive peer relationships are less likely to report any type of bullying experiences (Burton et al. 2013).

According to previous findings, adolescents who were bullied in either offline or online circumstances have higher levels of depression and lower self-esteem than those without any bullying experiences (Gámez-Guadix et al. 2013; Patchin and Hinduja 2010). However, there is no consensus on whether psychiatric symptoms (e.g., depression, anxiety) and problematic maladaptive behaviors (e.g., substance use, problematic Internet use) are precursors or consequences of bullying victimization. A number of studies have treated depressive symptoms and self-esteem as predictors of victimization (e.g., Chang et al. 2015; Olenik-Shemesh et al. 2012), whereas other studies have considered them as outcomes of victimization (Patchin and Hinduja 2010; Perren et al. 2010). However, according to a longitudinal study by Gámez-Guadix et al. (2013), depression predicted cyberbullying victimization, and in turn, cyberbullying victimization exacerbated pre-existing depressive symptoms. In contrast, psychoactive substance use predicted an increased risk of cyberbullying victimization but cyberbullying victimization did not predict an increased use of psychoactive substances. Given these findings, the present study focused on the role of psychoactive substance use and problematic Internet use in traditional and cyberbullying victimization among a representative adolescent sample while also acknowledging the unquestionable importance of psychological correlates such as depressive symptoms and self-esteem in bullying research.

## Aim of the Study

Recently, there has been a growing research interest in the relationship between bullying victimization and various addictive behaviors (Gámez-Guadix et al. 2013; Litwiller and Brausch 2013). The majority of previous studies have examined substance-related addictions and technology-related addictions separately in relation to traditional bullying and cyberbullying behaviors (Eksi 2012; Radliff et al. 2012), despite recent studies suggesting that problematic maladaptive behaviors might be general predictors of victimization (Gámez-Guadix et al. 2013; Jessor 1991). Therefore, the present study examined the extent to which there are specific potentially compulsive and/or addictive offline behaviors (i.e., psychoactive substance use) and online behaviors (i.e., excessive use of the Internet and time spent on social networking sites) that might be associated with either traditional bullying or cyberbullying victimization. According to the hypotheses of both Gámez-Guadix et al. (2013) and Jessor (1991), it was expected that potentially compulsive and/or addictive offline and online problem behaviors would predict victimization irrespective of medium (i.e., offline or online). Furthermore, the present research also investigated the role of gender and school type as well as social support in bullying victimization among a nationally representative sample.

## Methods

### Participants and Procedure

Data were collected within the European School Survey Project on Alcohol and Other Drugs (ESPAD; Király et al. 2015). The primary purpose of this international collaboration (which first began in 1995) was to gather data concerning the consumption of tobacco, alcohol, and illicit drugs among adolescents. In March 2015, the fifth wave of data collection was conducted, and the Hungarian section of the questionnaire also included a short section focusing on traditional bullying and cyberbullying victimization, in addition to Internet use habits. The target population of the present study comprised adolescents aged 16 years; therefore, the survey was administered to students across two grades (i.e., 9th and 10th). In order to obtain a nationally representative sample, a homogenous stratified random sampling method was applied based on the following variables: region (central/western/eastern Hungary), grade (9th and 10th), and school type (secondary general, secondary vocational, vocational schools). The self-report survey was completed anonymously by each student who was present in the classroom at the time of the data collection. The overall refusal rate was 7% in the primary sampling unit (i.e., classes), and data were consequently weighted due to skewed results produced by non-response bias. In order to yield a composition of participants that matched with the sampling frame, data were weighted by strata with the matrix weighting method suggested by the Education Information System 2014/2015 (KIR-STAT; Elekes 2012).

A total of 6664 adolescents participated in this survey (50.94% male). Participants who did not answer to either of the two questions regarding their bullying experiences (traditional or cyberbullying) were excluded from the analysis (*n* = 427). Thus, the final sample comprised 6237 adolescents (51.13% male). Age ranged from 15 to 22 years (*M*_age_ = 16.62, SD = 0.95). This relatively wide age range was attributed to the presence of older 9th and 10th grade students who might have repeated one or more school years after failing in one or more subjects taken. Approximately half of the adolescents were 9th graders (53.62%) and 46.38% were 10th graders. Almost half studied in general secondary school (43.34%), whereas 33.43% studied in secondary vocational school and 23.24% in vocational school. The study was approved by the Scientific Ethical Committee of Corvinus University of Budapest and was carried out in accordance with the Helsinki declaration.

### Measures

#### Sociodemographic Data

Data regarding gender, age, school type, grade, and region were collected.

#### Traditional Bullying and Cyberbullying Victimization

Bullying victimization was assessed using three items. One item focused on traditional bullying victimization and two items focused on different forms of cyberbullying experiences. First, all participants read a common definition of bullying based on the description of Shaw et al. (2013): “A student is bullied when a student or a group of students do or say rude and harassing things to him/her. Bullying also includes cases when someone is repeatedly called names in a nasty way, being teased, or being left out on purpose. It is not bullying when two young people who are as strong as each other argue or fight, or teasing is done in a friendly, playful way” (pp. 35). Second, participants were asked to report their bullying experiences (“In the last few months, how often were you bullied that way in the school?”) on a 5-point Likert scale (1 = I was not bullied by other students in the last few months, 2 = it happened 1–2 times, 3 = 2–3 times per month, 4 = weekly, 5 = more than once a week). Third, participants were asked to indicate how often they had been bullied by others in the following ways: (i) “Someone sent me insulting and harassing messages via chat program, e-mail, or SMS; posted such messages on my social networking site wall; or created a website to make fun of me”; and (ii) “Someone took embarrassing pictures of me and posted them online without my permission”*.* These forms of cyberbullying were chosen based on prior studies reporting that the most common forms of cyberbullying victimization were found to be receiving harassing messages and sharing embarrassing pictures online without permission (e.g., Patchin and Hinduja 2010; Slonje and Smith 2008). The same response format was used for these questions just as for traditional bullying victimization. The two cyberbullying victimization items were significantly correlated with each other (*r* = 0.34; *p* < 0.001). Similar to previous studies (e.g., Kowalski and Limber 2013; Patchin and Hinduja 2010), participants who were bullied at least 2–3 times per month were considered traditional bullying victims, and those who reported that they experienced either form of cyberbullying at least 2–3 times per month were considered cyberbullying victims.

#### Social Support

Participants were asked to indicate how often they felt the following statements were true of them: (i) “I can get understanding and caring from my mother and/or father easily”; and (ii) “I can get emotional support from my mother and/or father easily”. To assess the received emotional support from a best friend, the same questions were asked replacing “mother and/or father” with “best friend,” and participants indicated their level of agreement with the statements on a 5-point Likert scale ranging from 1 (almost always) to 5 (almost never). The two variables were created by averaging the scores of parental support and best friend’s support. For the sake of clarity, scores were reversed during the analysis, with higher scores indicating higher social support. The questions were significantly correlated with each other (*r*_parents_ = 0.81, *p* < 0.01; *r*_best friend_ = 0.86, *p* < 0.01). However, the constructed items related weakly to each other (*r* = 0.32, *p* < 0.001). The Cronbach’s alpha was 0.89 for parental support and 0.92 for best friend’s support.

#### Psychoactive Substance Use

Problem offline behaviors included tobacco, alcohol, and drug consumption. First, participants were asked “How many times did you smoke in the last 30 days?” (0 = not at all, 1 = less than one cigarette per week, 2 = less than one cigarette per day, 3 = 1–5 cigarettes per day, 4 = 6–10 cigarettes per day, 5 = 11–20 cigarettes per day, 6 = more than 20 cigarettes per day). In the present analysis, this variable was dichotomized in the following way: 0 = did not smoke at all, 1 = smoked any cigarettes (from 1 to 6). Second, they were asked to indicate how many times they consumed alcohol in the last 30 days (0 = 0 times, 1 = 1–2 times, 2 = 3–5 times, 3 = 6–9 times, 4 = 10–19 times, 5 = 20–39 times, 6 = 40 or more times). This variable was linearized by using the middle points of the values and was treated as a continuous variable in the data analysis. Finally, students were asked to indicate how many times they used a number of illicit psychoactive substances in the last 12 months (i.e., marijuana, ecstasy, amphetamines, methamphetamines, cocaine, crack, and inhalants). The same response scale was used as for alcohol consumption, but the variables were dichotomized in the following way: 0 = did not use the respective drug and 1 = used the respective drug at least 1–2 times in the last 12 months. The drug use variable was created by summing the seven items and was treated as a continuous variable in the analysis.

#### Internet Use

Information regarding Internet use habits was gathered in terms of problematic Internet use and time spent on social networking sites, which has been identified as an increasingly risky online activity in studies focusing on cyber-victimization (Mesch 2009; Sampasa-Kanyinga and Hamilton 2015). Problematic Internet use was assessed with the 6-item version of the Problematic Internet Use Questionnaire (PIUQ-6; Demetrovics et al. 2016). A typical item is “How often do you feel tense, irritated, or stressed if you cannot use the Internet for as long as you want to?” Participants were asked to respond to these six questions on a 5-point Likert scale ranging from 1 (never) to 5 (always). The Cronbach’s alpha was 0.79.

Participants’ weekly time spent on social networking sites was assessed by two questions: (i) “How many days did you use the Internet for social networking in the last 7 days?” (from 1 = 1 day to 7 = 7 days, and 0 indicated “I did not use”); and (ii) “On an average day, how many hours did you use the Internet for social networking in the last 30 days?”(1 = 0 h, 2 = less than half an hour, 3 = 1 h, 4 = 2–3 h, 5 = 4–5 h, 6 = more than 6 h). A computed variable was created by combining these two question types. Given that a considerable proportion of the participants (29.80%) spent more than 6 h per day on social networking sites, this variable was dichotomized in the following way: 0 = spent 6 or less hours per day on social networking sites and 1 = spent more than 6 h per day on social networking sites.

#### Psychological Correlates

Self-esteem was assessed using the Hungarian version of the Rosenberg Self-Esteem Scale (RSES-HU; Rosenberg 1986; Urbán et al. 2014). The RSES-HU comprises 10 items (e.g., “I feel that I have a number of good qualities”), which assess global self-esteem on a 4-point Likert scale ranging from 1 (strongly disagree) to 4 (strongly agree), with higher scores indicating higher self-esteem. The Cronbach’s alpha was 0.86 in the present sample. Depressive symptoms were assessed using the short, 6-item version of the Center for Epidemiological Studies Depression Scale (CES-D; Radloff 1977). The statements were translated into Hungarian following the protocol of Beaton et al. (2000). A typical item is “You had trouble keeping your mind on what you were doing.” Participants rate each item on a 4-point Likert scale (from 1 = almost never to 4 = almost always), with higher scores indicating higher levels of depressive symptoms. The Cronbach’s alpha for both scales was 0.85.

### Statistical Analysis

Data analysis was conducted using SPSS version 22.0 (IBM SPSS Inc., Chicago, IL) and Mplus 8.0 (Muthén and Muthén 1998–2017). Data were weighted to reflect a nationally representative sample of 9th and 10th grade adolescents. For comparisons across different victim groups, effect size indices (Cohen’s *d*) were calculated with 0.1 being interpreted as a small effect, 0.3 as a medium effect, and 0.5 as a large effect (Field 2009). To investigate the role of social support and addictive behaviors in traditional bullying and cyberbullying victimization, multivariate probit regression model with weighted least squares parameter estimation (weighted least squares–mean and variance adjusted, WLSMV) within a structural equation modeling (SEM) framework was conducted in which traditional and cyberbullying victimization (as binary variables) were entered as dependent variables in one model, whereas the independent variables were (i) gender, school type, traditional bullying, or cyberbullying victimization; (ii) parental and best friend’s support; (iii) tobacco, alcohol, and drug consumption; and (iv) problematic Internet use and weekly social networking time.

## Results

### Descriptive Statistics

Descriptive statistics of students’ victimization in different bullying forms are presented in Table 1. A total of 832 adolescents (13.33%) reported that they were bullied at school in the past few months. Furthermore, 985 (15.80%) received insulting messages online and 705 (11.30%) reported that someone took embarrassing pictures of them and posted them online without permission.

**Table 1:** Descriptive statistics of traditional bullying and cyberbullying victimization (*N* = 6237)

	Traditional bullying	Cyberbullying (messages)	Cyberbullying (photos)
Not bullied	5406 (86.67%)	5252 (84.20%)	5531 (88.68%)
1–2 times	574 (9.20%)	850 (13.63%)	579 (9.29%)
2–3 times per month	85 (1.36%)	73 (1.17%)	79 (1.27%)
About weekly	84 (1.34%)	28 (0.44%)	18 (0.30%)
More than once a week	89 (1.43%)	34 (0.54%)	29 (0.47%)

The number of participants is reported for the frequencies of victimization with the respective percentages in parenthesis

For further analysis, traditional bullying victims (i.e., those who were bullied at school at least 2–3 times in the last few months), cyberbullying victims (i.e., those who were harassed online at least 2–3 times in the last few months), participants who were not victimized, and those who experienced both forms of bullying (i.e., offline and online) were compared. The results are presented in Table 2.

**Table 2:** Group comparisons based on different types of victimization (*TB* victim of traditional bullying, *CB* victim of cyberbullying, *CB* and *TB* victim of both forms)

		Form of victimization				
	Total (*N* = 6237)	Not victimized (*n* = 5805)	TB victim (*n* = 204)	CB victim (*n* = 174)	CB and TB victim (*n* = 54)	Chi-square/F/Welch
Gender						
Male	3127 (50.13%)	2910 (51.13%)a	94 (46.08%)a	95 (54.29%)a	28 (51.85%)a	2.61
School type						
Secondary general	2703 (43.34%)	2559 (44.08%)a	81 (39.90%)ac	47 (27.01%)b	16 (29.09%)bc	41.86***
Secondary vocational	2085 (33.43%)	1941 (33.44%)a	66 (32.51%)a	57 (32.76%)a	21 (38.18%)a	
Vocational	1449 (23.24%)	1305 (22.48%)a	57 (27.59%)a	70 (40.23%)b	17 (32.72%)ab	
Social support						
Parents	4.22 (1.04)	4.25 (1.02)a	3.95 (1.24)b	3.77 (1.17)b	3.55 (1.44)b	16.02***
Best friend	4.18 (1.05)	4.20 (1.04)a	3.90 (1.24)bc	4.01 (1.08)ac	3.70 (1.30)bc	7.50***
Psychoactive substance use						
Tobacco consumption	2167 (34.79%)	1983 (34.22%)a	70 (34.31%)a	89 (51.15%)b	24 (45.28%)ab	23.95***
Alcohol (number of drinks)	2.86 (4.89)	2.76 (4.70)a	3.03 (5.76)a	4.91 (7.06)b	5.75 (9.44)b	7.06***
Drugs (range of types used)	0.22 (0.73)	0.19 (0.64)a	0.36 (1.18)b	0.82 (1.66)c	0.79 (1.59)c	11.54***
Internet use						
Problematic use	1.85 (0.73)	1.83 (0.71)a	2.07 (0.85)b	2.33 (0.89)c	2.34 (1.13)bc	25.35***
Frequent SNS use	1824 (29.91%)	1677 (29.53%)a	64 (32.00%)ab	66 (38.82%)b	18 (34.61%)ab	7.80*
Psychological correlates						
Depressive symptoms	1.92 (0.61)	1.89 (0.59)a	2.19 (0.69)b	2.38 (0.71)c	2.57 (0.89)c	47.86***
Self-esteem	2.73 (0.61)	2.76 (0.60)a	2.34 (0.60)b	2.38 (0.64)b	2.30 (0.55)b	59.19***

The number of participants is reported for gender, school type, tobacco, and SNS time with the respective percentages in parenthesis, whereas means and standard deviations (in parenthesis) are reported for social support, alcohol and drug use, Internet use, and psychological correlates variables

Tobacco consumption was anchored at 0 = did not smoke any cigarettes in the last 30 days and 1 = smoked cigarettes in the last 30 days. The range of drug use was between 0 and 7, in which 0 indicated the lack of experiment with any drugs, and 7 indicated experiences with all types of drugs described in the present study. SNS use was anchored at 0 = used social networking sites for less than 42 h per week and 1 = used social networking sites for more than 42 h per week

Different letters (a, b, c) in the same row represent significant (*p* < 0.05) difference between the percentages and mean scores, whereas the same letters in the same row represent non-significant difference between the percentages and mean scores according to the paired chi-square tests and post hoc Tukey or Scheffe test of one-way ANOVA

*p < 0.05; **p < 0.01; ***p < 0.001

No significant gender differences were observed regarding victimization in different types of bullying (*χ* ^2^(3) = 2.61, *p* = 0.46). In contrast, significant differences were found in the form of victimization across different school types. Traditional bullying was more prevalent than cyberbullying among secondary general students, whereas cyberbullying was more frequently reported among vocational school students than traditional bullying (*χ* ^2^(6) = 41.86, *p* < 0.001). The highest perceived parental and best friend’s support was yielded by those who were not victimized in any forms of bullying, whereas the lowest scores were obtained by students who experienced both forms of bullying. Although the difference between these two groups was significant, the effect sizes were relatively small (Welch_parental_(153) = 16.02, *p* < 0.001, *d* = 0.11; Welch_best friend_(170) = 7.50, *p* < 0.001, *d* = 0.07). Furthermore, no significant difference was found between traditional bullying and cyberbullying victims in social support variables.

Interestingly, the highest frequency of psychoactive substance use was reported by cyberbullying victims. The difference between traditional and cyberbullying victims in tobacco, alcohol, and illicit drug consumption was significant with small to moderate effect sizes (*χ* ^2^_tobacco_(1) = 10.92, *p* = 0.001, Phi = 0.17; *t*_alcohol_(332) = 2.79, *p* = 0.006, *d* = 0.29; *t*_drug_(299) = 3.05, *p* = 0.002, *d* = 0.32). Cyberbullying victims scored significantly higher on the problematic use scale than those students who did not report any cyberbullying experiences, although the effect size was small (Welch(170) = 25.35, *p* < 0.001, *d* = 0.14). Furthermore, cyberbullying victims spent more time on social networking sites compared to those students who were not bullied either offline or online (*χ* ^2^(1) *=* 6.82, *p* = 0.01, Phi = 0.03).

Regarding psychological correlates, adolescents who reported being both traditional bullying and cyberbullying victims reported greater levels of depressive symptoms and lower levels of self-esteem compared to students who were not victimized in any forms of bullying. Group differences appeared to be significant, but the effect sizes were relatively small (Welch_depressive symptoms_(172) = 47.86, *p* < 0.001, *d* = 0.19; F_self-esteem_(6042) = 59.19, *p* < 0.001, *d* = 0.17). Although no difference was found between traditional bullying and cyberbullying victims regarding self-esteem, students with cyberbullying experiences reported greater levels of depressive symptoms compared to students who experienced only traditional bullying or neither type of bullying.

### Predictors of Traditional Bullying and Cyberbullying Victimization

To analyze the possible convergent and divergent predictor variables of traditional bullying and cyberbullying victimization, multivariate regression was conducted (see Table 3). Based on previous analyses, the study variables across different school types demonstrated that secondary general and secondary vocational school students shared more similarities with each other than with vocational school students. Therefore, the school type variable was dichotomized for further analysis (0 = vocational school (3-year education) and 1 = secondary high schools (4-year education)).

**Table 3:** Multivariate probit regression model predicting traditional bullying and cyberbullying victimization

	Traditional bullying victimization	Cyberbullying victimization
	B (SE)	B (SE)
Demographic characteristics		
Female	0.101 (0.068)	0.050 (0.070)
Vocational school student	− 0.048 (0.078)	− 0.184 (0.077)*
Social support		
Parents	− 0.068 (0.028)*	− 0.103 (0.030)*
Best friend	− 0.092 (0.028)**	− 0.029 (0.032)
Psychoactive substance use		
Tobacco consumption	− 0.037 (0.071)	0.002 (0.074)
Alcohol (number of drinks)	− 0.004 (0.007)	0.012 (0.006)
Drugs (range of type used)	0.119 (0.038)***	0.153 (0.037)***
Internet use		
Problematic use	0.196 (0.038)***	0.245 (0.040)***
Frequent SNS use	− 0.032 (0.070)	0.075 (0.075)
*R* ^2^	0.048	0.086

The model is estimated with WLSMV estimator in Mplus 8.0^.^ In the model, the estimated tetrachoric correlation between TB and CB is 0.438***

*TB* traditional bullying, *CB* cyberbullying

*p < 0.05; **p < 0.01; ***p < 0.001

The regression model revealed no gender difference in either traditional bullying or cyberbullying victimization, meaning that boys and girls were equally likely to report bullying experiences. In contrast, school type was found to be a significant predictor of cyberbullying experiences. Vocational school students were more likely to report online victimization compared to high school students. However, no relationship was found between school type and traditional bullying victimization. Higher perceived parental support was negatively associated with traditional bullying and cyberbullying victimization. Students reporting higher perceived best friend’s support were less likely to be victimized in traditional bullying, but peer support was not protective against cyberbullying victimization.

In relation to psychoactive substance use, illicit drug use was a significant predictor of victimization in both bullying forms, whereas smoking cigarettes and alcohol consumption showed no association with either form of victimization. Problematic Internet use was related to an increased risk of victimization in both traditional bullying and cyberbullying. However, no association was found between social networking sites use and victimization.

The crude tetrachoric correlation between the two types of bullying was 0.503. However, after adjusting for the predictor variables in the model, the estimated tetrachoric correlation changed only slightly (*r* = 0.438). In sum, the strongest predictors of victimization were problematic Internet use and drug use. Furthermore, traditional bullying victimization was associated with low best friend’s support, whereas cyberbullying victimization was associated with low parental support. Moreover, vocational school students were more likely to report cyberbullying victimization compared to students of other school types. However, the variance explained by these variables was very small in both cases. The final model explained only 4.8% of the total variance of traditional bullying and 8.6% of cyberbullying. This suggests that other variables need to be considered in future research that aims to identify significant predictors of victimization in traditional bullying and cyberbullying.

## Discussion

Previous research has proposed that problematic maladaptive behavior of adolescents may increase the risk of peer aggression, which in turn may increase the risk of victimization (Gámez-Guadix et al. 2013; Jessor 1991). Using a nationally representative sample of Hungarian middle school students, the present study provided further empirical evidence for this hypothesis by demonstrating that drug use and problematic Internet use are weakly but significantly associated with traditional bullying and cyberbullying victimization. However, these potentially compulsive and/or addictive behaviors had limited explanatory power for predicting bullying victimization in either case. These results suggest that potential offline and online compulsive and/or addictive behaviors are associated with both bullying forms, but psychoactive substance use and excessive Internet use do not appear to be key variables underlying bullying victimization.

In the present study, 4.14% of students reported being bullied repeatedly offline and 3.66% online in the past few months preceding the data collection. Additionally, 21% of students who reported being victimized in traditional bullying also reported being victimized in cyberbullying, whereas 24% of cyberbullying victims also reported being bullied by peers at school. These results echo those studies using similar time periods and adolescent samples. For instance, Sittichai (2014) found that 6% of 14–17-year-old students were bullied frequently over the past couple of months. In another study, 7.1% of 10–13-year-old students were bullied online frequently in the last 3 months (Price et al. 2013). The relatively higher prevalence rates in these studies compared to the prevalence rates in the present study may be due to the wider age range. Indeed, it has been found that traditional bullying victimization (Scheithauer et al. 2006) and cyberbullying victimization (Smith et al. 2008) are more prevalent among younger adolescents than among older ones. Furthermore, the prevalence of traditional bullying victimization was higher compared to cyberbullying victimization, consistent with previous studies which have concluded that cyberbullying has yet to exceed the prevalence of traditional bullying experiences (Sabella et al. 2013).

Similar to a number of previous studies (e.g., Bauman et al. 2013; Hinduja and Patchin 2008), no gender differences were found in either traditional bullying or cyberbullying victimization. Regarding school types, vocational school students were more likely to report cyberbullying experiences compared to students of other school types. One possible explanation may be that vocational school students had the highest scores on the Problematic Internet Use Questionnaire (PIUQ), and problematic Internet use has been associated with the increased risk of cyberbullying victimization in previous studies (Casas et al. 2013; Chang et al. 2015). These studies argued that problematic Internet users are characterized by increased irritability which could lead to aggressive behaviors (e.g., insulting other users). Such a result draws attention to risky Internet use among vocational students. Future research should investigate the role of this educational context in maladaptive problem behaviors associated with cyberbullying victimization.

In relation to victim groups, the highest level of depressive symptoms and the lowest levels of self-esteem were reported by those students who experienced both traditional bullying and cyberbullying. This result confirms previous findings (Hinduja and Patchin 2010; Nixon 2014). Although traditional bullying victimization was more prevalent among students than cyberbullying, those who reported being bullied online reported greater levels of depressive symptoms and psychoactive substance use compared to those who were only bullied at school. This finding is similar to the study of Smith et al. (2008) in which two cyberbullying forms (i.e., text messages, and using photos or videos without permission) had a greater emotional impact on victims than more traditional bullying forms.

With regard to social support, low levels of perceived best friend’s support predicted traditional bullying victimization, whereas low parental support was associated with an increased risk of victimization in both forms of bullying. Perhaps unsurprisingly, these results suggest that the presence of a supportive best friend may be a protective factor against schoolyard bullying where harassment occurs, whereas parental care and monitoring appear to have an important role in the prevention of any type of bullying experiences.

The potentially problematic behaviors of psychoactive substance use and Internet use were associated with an increased risk of victimization in both traditional bullying and cyberbullying. This result is also in line with previous findings (Casas et al. 2013; Chang et al. 2015). However, the explained variance of these factors in bullying victimization was extremely limited (i.e., 4.8–8.6% of the total variance), similar to that reported by Hinduja and Patchin (2008) who found that traditional bullying experiences and problem behaviors (e.g., substance use) explained only 1–5% of variation in cyberbullying victimization.

This study is not without its limitations. Due to the cross-sectional nature of the study, the direction of associations could not be determined. Similarly, the present authors treated social support and problem behavior variables as predictors in the hypothesized models, whereas psychological correlates were excluded from the regression analysis as they were considered as outcomes, similar to several prior studies (e.g., Chang et al. 2015; Perren et al. 2010). However, alternative models should be considered in future studies. Another limitation was the relatively narrow forms of cyberbullying victimization that were assessed (i.e., only two items); therefore, a wider range of harassment types should be assessed in future research. Finally, considering the low explanatory power of the tested models, other factors should be considered when examining the associates of cyberbullying victimization.

The primary purpose of the present study was to offer a more nuanced understanding of the associations of offline and online problem behaviors with both traditional and cyberbullying victimization, and enhance the growing body of literature on the relationship between various substance and behavioral addictions and bullying. Based on the findings of the present study, it is concluded that traditional bullying victimization was associated with cyberbullying victimization. Furthermore, psychoactive substance use and problematic Internet use predicted both online and offline bullying. However, the low explanatory power of the tested model suggests the importance of other factors that are likely to be stronger predictors of traditional bullying and cyberbullying victimization, and it is recommended that other factors should be explored in future research.
